# Recent Advances in a Polydopamine-Mediated Antimicrobial Adhesion System

**DOI:** 10.3389/fmicb.2020.607099

**Published:** 2021-01-12

**Authors:** Indu Singh, Gagan Dhawan, Seema Gupta, Pradeep Kumar

**Affiliations:** ^1^Acharya Narendra Dev College, University of Delhi, Delhi, India; ^2^Nucleic Acids Research Laboratory, CSIR-Institute of Genomics and Integrative Biology, Delhi, India

**Keywords:** biofilm, polymerization, ROS, polydopamine, antimicrobial, surface coating

## Abstract

The drug resistance developed by bacteria during antibiotic treatment has been a call to action for researchers and scientists across the globe, as bacteria and fungi develop ever increasing resistance to current drugs. Innovative antimicrobial/antibacterial materials and coatings to combat such infections have become a priority, as many infections are caused by indwelling implants (e.g., catheters) as well as improving postsurgical function and outcomes. Pathogenic microorganisms that can exist either in planktonic form or as biofilms in water-carrying pipelines are one of the sources responsible for causing water-borne infections. To combat this, researchers have developed nanotextured surfaces with bactericidal properties mirroring the topographical features of some natural antibacterial materials. Protein-based adhesives, secreted by marine mussels, contain a catecholic amino acid, 3,4-dihydroxyphenylalanine (DOPA), which, in the presence of lysine amino acid, empowers with the ability to anchor them to various surfaces in both wet and saline habitats. Inspired by these features, a novel coating material derived from a catechol derivative, dopamine, known as polydopamine (PDA), has been designed and developed with the ability to adhere to almost all kinds of substrates. Looking at the immense potential of PDA, this review article offers an overview of the recent growth in the field of PDA and its derivatives, especially focusing the promising applications as antibacterial nanocoatings and discussing various antimicrobial mechanisms including reactive oxygen species-mediated antimicrobial properties.

## Introduction

A considerable number of catecholic residues similar to those found in the mussel foot proteins (mytilus foot proteins, MFP-3 and 5) and melanin have attracted the attention of researchers around the globe. A plethora of articles/reviews has been presented on the structure, reaction mechanism, and biomedical and biotechnological applications of materials containing these functionalities (Solano, 2014, 2017; Ahn, 2017; Ball, 2017; Forooshani and Lee, 2017; Maier and Butler, 2017; Rahimnejad and Zhong, 2017; Moulay, 2018; Park et al., 2019). Polydopamine (PDA) is one such material and is an extremely interesting polymer, ennobled with unique features – such as adherence to all types of surfaces even under water, a characteristic attributed to the catechol moieties in its monomeric building blocks. The polymer, reaped by dopamine oxidation, contains indole and dopamine units in various oxidation states and also, to a smaller extent, pyrroles. The oxidized o-quinone and o-hydroquinone groups in its chemical structure underlie the complex oxidation chemical characteristics of PDA (Lynge et al., 2011; Jia et al., 2019). Further, it has been shown that, during oxidation of dopamine, reactive oxygen species are generated, which act as bactericidal against both Gram-positive and Gram-negative bacteria (Lynge et al., 2011). Hence, the coating of such materials on the surfaces affects the growth and survival of microorganisms. Work has also indicated that PDA has the potential to reduce the *in vivo* toxicity of biomaterials, which come in contact with the tissues or blood and suggest it to be a versatile platform (Hong et al., 2011).

Since the method reported by Lee et al. (2007) for dopamine self-polymerization to form thin films, surface-adherent, multifunctional PDA coatings through simple dip-coating of a wide-range of inorganic and organic materials, many PDA-based nanomaterials have been generated. In a recent study, it has been shown that PDA nanoparticles synthesized by laccase catalysis were more stable in strongly alkaline and acidic solutions than those synthesized by more traditional chemical routes (Li et al., 2018), although chemical oxidation processes are more cost effective for mass production and reproducibility. Various additives incorporated during synthesis can tune material properties via covalent cross-linking or physical entrapment and through non-covalent interactions (Ang et al., 2016; Chen et al., 2018; Wang et al., 2018), such as cation–π interactions (Hong et al., 2018). In a similar vein, chemically reactive nanoparticles can act as core templates for multifunctional use, such as coatings on the surface of other template/core materials (Lin et al., 2015; Hong et al., 2017). A review article by Ball has described the synthesis methods that yield PDA nanoparticles in the absence/presence of templating agents along with the use of thin PDA layers on nanoparticles or nanotubes (Ball, 2017). The results of synthesis in the presence of templating agent indicate a strong correlation between the catecholamine and the templating molecules with awaited investigation on the nature and the strength of interactions (Ball, 2018).

## Role of Covalent and Non-Covalent Interactions in Polydopamine Surface Chemistry

Despite the research being carried out, the chemical basis of the amazing underwater adhesion properties of PDA remains unclear. Mussel adhesiveness has persuaded scientists to explore the self-polymerization of dopamine under alkaline conditions. The solution to this puzzle could provide a universal type of coating for polymers, metals, and ceramics irrespective of their physical and chemical properties. The polymerized layer, enriched with catechol groups, can immobilize primary amine or thiol-based biomolecules through a simple dipping process. In one of the studies, Hong et al. (2012) have demonstrated that a small amount of free dopamine left physically entrapped in synthesized PDA does not exhibit cytotoxicity, as it is rarely released due to strong non-covalent π–π interactions with aromatic rings.

**FIGURE 1:**
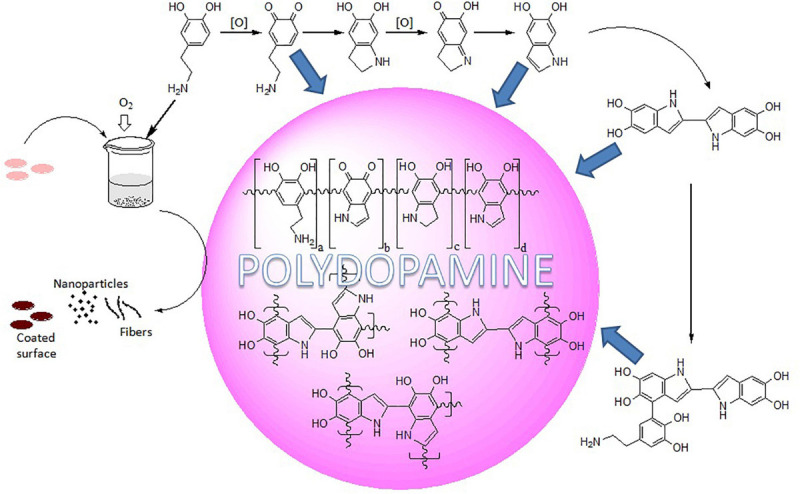
Schematic representation of oxidative self-polymerization of dopamine.

Work to understand the PDA structure is still under deliberation and suggests that it is composed of dihydroxyindole, indoledione, and dopamine units, covalently linked (Figure 1). Further verification of the presence of covalently linked dihydroxyindole and indoledione units between their aromatic rings leading to different degrees of unsaturation has been suggested by Liebscher et al. (2013). The work by Alfieri et al. (2017) has concluded that PDA is different from 5,6-dihydroxyindole and melanin, and PDA film formation requires amine-containing structural components for adhesion and film-forming properties. Further, the group has noticed that PDA film formation involves competition/dynamic interactions among the intermolecular amine–quinone condensation processes, while film deposition accelerates with quinone-forming oxidants and depends on dopamine concentration and becomes practically negligible below 1 mM dopamine (Alfieri et al., 2018). The catechol groups of PDA have the ability to reduce noble metal salts (Ag^+^, Au^3+^) to metal nanoparticles and immobilize the nanoparticles within the scaffolds, thereby preventing aggregation or leaching (Holten-Andersen et al., 2009). A review by Ho and Ding (Ho and Ding, 2014) has highlighted the probable adhesion mechanisms, chemical/physical properties, and applications of PDA. Liebscher has presented an overview on the chemistry and properties of PDA and its analogs (Liebscher, 2019). Among all the rationale for the overall mechanism for the polydopamine formation, two different pathways have also been suggested: self-assembled (dopamine)_2_/5,6-dihydroxyindole (DHI) trimmers, assembled via quadrupole–quadrupole, hydrogen bonding, and π–π interactions, and dopamine–DHI–DHI conjugates, formed via covalent bond (Hong et al., 2012). However, all the views conclude that the insoluble biopolymer PDA system, formed by autoxidation of the catecholamine neurotransmitter dopamine, encompasses non-cyclized dopamine units, 5,6-dihydroxyindole (DHI), indole-5,6-quinone (IQ), and their oligomers as the main structural units (Barclay et al., 2017).

As PDA and eumelanins are found in various parts of the human body, they possess excellent physicochemical properties and provide good biocompatibility (d’Ischia et al., 2014). Hence, PDA holds considerable promise for integration with biological tissues. Polydopamine coatings are accepted as the foremost outfit for functionalization of practically any kind of material surface. The absence of strong π-conjugated system of catechol in DHI is caused by protonated uncyclized amines supporting the formation of polydopamine (Lee et al., 2019). Several factors, viz., initial dopamine concentration, pH, reaction temperature, and choice of buffer and oxidant, are reported to play important roles during the preparation of PDA coatings, and, subsequently, the physical properties of PDA coatings control the surface characteristics, which, in turn, might significantly alter the antibacterial activities (Ding et al., 2016).

Surface wettability is an important property based on surface energy and morphology of the material that addresses adhesion. The hydrophobic surface exhibits stronger adhesion with the PDA coatings compared to the hydrophilic surfaces, as a “hydrophobic depletion layer” on the hydrophobic substrate is suggested to enhance the PDA adhesion by allowing an intimate contact between PDA and the substrate (Zhang et al., 2017). The hydrophilic behavior of PDA due to the presence of polar functional groups supports cell attachment and growth. Various co-deposition processes have been suggested to address the insufficient hydrophilicity in PDA (Qiu et al., 2018).

## Role of Michael Addition/Schiff Base Reactions

**FIGURE 2:**
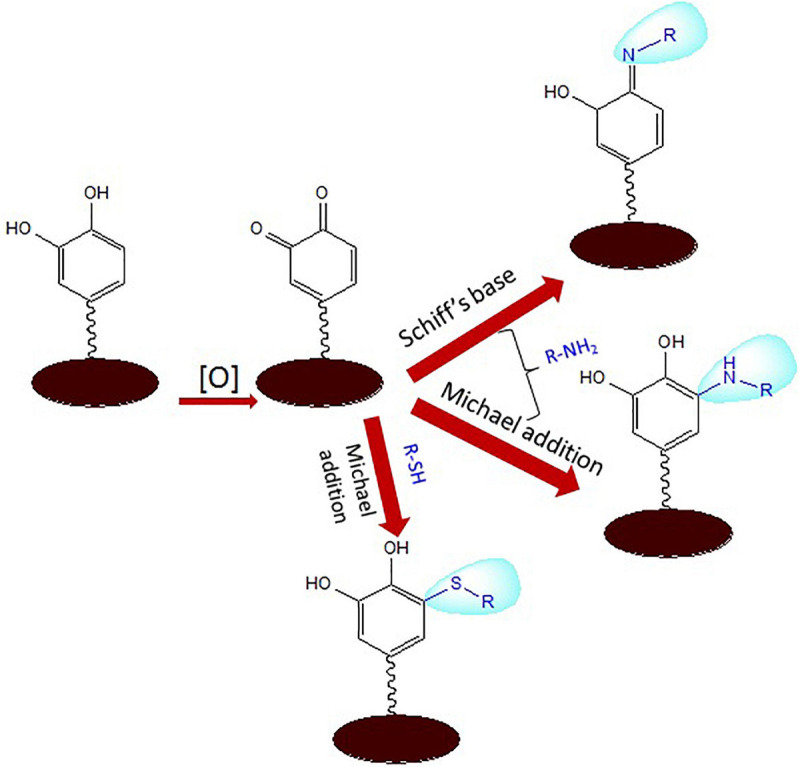
Schiff base and Michael addition reactions of aminated and thiolated ligands with reactive functionalities of polydopamine (PDA).

Polydopamine is moderately hydrophilic due to the large volumes of catechol, quinine, and amine groups. Reactants bearing functionalities such as amines and thiols can target the diketone or catechol groups exposed on the PDA surface via aza- or thio-Michael addition and Schiff base reactions, which can tune the surface properties of the polymer (Figure 2). Hence, PDA coatings can play the role as anchor to tether biopolymers consisting of thiols and amines (Lee et al., 2009; Huang et al., 2015; Liu and Huang, 2016; Tang et al., 2018; Yang et al., 2019). This has allowed immobilization of oligonucleotide-based aptamers and molecular probes and multifunctional nanoparticles for potential applications in nanomedicine and biosensors (Wang C. et al., 2016; Zheng et al., 2018; Jia et al., 2019). The phenolic groups of PDA have been reported to enhance the nucleation sites in the carbon dot formation to fabricate fluorescent PDA-passivated N-doped carbon dots (Bai et al., 2018).

## Broad Spectrum of Polydopamine Nanoparticles as Biomaterials/Nanocoatings

Polydopamine finds numerous applications even as stable suspension of nanoparticles (NPs), particularly as biomaterials. The use of PDA/hybrids in various fields, e.g., converting light to heat for cancer treatment by hyperthermia (Liu F. et al., 2015; Dong et al., 2016; Wang X. et al., 2016; Li Y. et al., 2017; Nieto et al., 2018; Tian and Lei, 2019), catalytic therapy for tumors (Zhu et al., 2019), and for sensing (Kong et al., 2016; Wang Z. et al., 2017; Zhao et al., 2017; Gu et al., 2018), as well as PDA NPs loaded scaffolds in the field of tissue engineering (Deng et al., 2019), are all worth mentioning. A review article that highlights the progress in fluorescent PDA nanomaterials has been published by Yang et al. (2020b). PDA nanoparticles as sensors (Jiao et al., 2017; Ma et al., 2017), theranostics (Dong et al., 2016), or for drug release (Li W.Q. et al., 2017; Wang X. et al., 2019)/gene delivery (Priyam et al., 2018) are exploited regularly; for example, the toxic nature of polyethylenimine could be suppressed during covalent conjugation with PDA for gene delivery (Priyam et al., 2018). A review by Xiong et al. (2019) has discussed in detail the developments of PDA nanocarriers to deliver photosensitizers through chemical conjugation, physical absorption, and encapsulation strategies. A review by Batul et al. (2017) has discussed about the developments made in the area of drug delivery, photothermal therapy, and bone and tissue engineering using polydopamine nanostructures.

Polydopamine nanoparticles have been observed to display synergistic therapeutic effects for combined chemotherapy and photothermal therapy for cancer (Zhu and Su, 2017). PDA in association with photosensitizers conjugated to hyaluronic acid complexed nanoparticles has been reported to allow tumor-specific photodynamic therapy with degradation of the hyaluronic acid by the tumor-localized intracellular enzymes releasing the photosensitizer nanoparticles (Han et al., 2016).

Similarly, calcium carbonate–polydopamine (CaCO_3_–PDA) composite hollow nanoparticles have been reported to offer a multifunctional theranostic nanoplatform. The pH-dependent nanoparticles, quenched by PDA, could be photoactivated only in the acidic environment of a tumor whereby they released the photosensitizer and displayed multimodal imaging capability due to strong affinity between metal ions and PDA and exhibit high antitumor PDT efficacy (Dong et al., 2018). Various metal ions have been loaded onto PDA nanoparticles for bioimaging and photothermal cancer therapy simultaneously (Miao et al., 2015; Ge et al., 2017; Feng et al., 2020; Liu et al., 2020). Photocatalytic organic nanoparticles composed of flavin-conjugated PDA NPs have been described to display xenobiotic-degrading enzyme analogous activity (Fruk and Crocker, 2019).

## Applications of Polydopamine Nanocoatings

Polydopamine coatings have been designed to enhance mucopenetration as well as cell uptake of NPs for mucosal drug delivery applications (Poinard et al., 2019). Mallinson et al., with the help of atomic force microscopy, suggested that PDA could be a useful coating to reduce interaction with proteins, which would otherwise lead to fouling (Mallinson et al., 2018). The hydrophilicity, aqueous durability under the physiological conditions, biocompatibility, as well as ease of functionalization make PDA nanocoatings apt candidates for numerous tissue engineering applications. PDA-assisted hydroxyapatite coating onto porous Ti_6_Al_4_V scaffolds has been reported to promote osteointegration and osteogenesis *in vivo* and could be useful for bone defect repair (Li et al., 2015). Ku and Park (2010) introduced PDA coatings for potential vascular tissue engineering applications (Ku and Park, 2010). It has been shown that PDA offers simple, effective, and inexpensive alternative for bone tissue engineering compared to traditional surface modification and tissue regeneration (Huang S. et al., 2016; Lee et al., 2017; Zhou et al., 2019). Madhurakkat Perikamana et al. (2015) have discussed in detail various techniques for versatile surface modification for tissue engineering.

## PDA as a Reactive Oxygen Species Scavenger

The redox activities of catechol groups, pro- and antioxidant, in mussel adhesive proteins are a potential treasure for clinical applications. Catechol oxidation by air generates H_2_O_2_ and O_2_^⋅^^–^ as reactive by-products. Forooshani et al. (2017) have reviewed the regulation of reactive oxygen species (ROS) and their effects in living system. The group further applied this redox chemistry of adhesive moiety to design microgels, which had the potential to generate H_2_O_2_ as and when needed for antimicrobial and antiviral applications (Meng et al., 2019). The antimicrobial property of PDA could also be accredited to the catechol moieties, which are released in a controlled manner and autoxidize in the presence of oxygen, forming semiquinone and quinone. The oxidation process generates ROS including two well-known disinfectants, superoxide anions (O^2–^) and H_2_O_2_ (Alfieri et al., 2017; Ryu et al., 2018). PDA is able to remove ROS that are generated during inflammatory responses. PDA nanoparticles have been shown to possess antioxidative properties to remove ROS and suppress ROS-induced inflammation in periodontal diseases without any side effects (Bao et al., 2018) and have been used effectively in the treatment of acute inflammation-induced injury (Zhao et al., 2018). PDA-coated hemoglobin nanoparticles showed oxidative protection of hemoglobin and antioxidative properties to remove ROS as well as reduce ROS generation besides exhibiting high oxygen affinity and low cytotoxicity (Wang Q. et al., 2017). Arginine-doped PDA has been shown to be a free-radical scavenger due to greater accessibility to free radicals and so has exhibited superior antioxidant performance than PDA-melanins (Yang et al., 2020a). A nanocomposite based on V_2_O_5_/PDA/MnO_2_ has been reported to exhibit an ability to remove intracellular reactive oxygen species and mimic intracellular antioxidant enzyme-based defense system flaunting potential for inflammation therapy (Huang Y. et al., 2016). Cu-loaded PDA coatings are being seen as a favorable platform for blood contact materials, which have the capability to catalyze the decomposition of S-nitrosothiols and release NO for a longer period thus maintaining the anticoagulant effects (Zhou et al., 2020).

## Applications of Polydopamine as Antibacterial Agent

**FIGURE 3:**
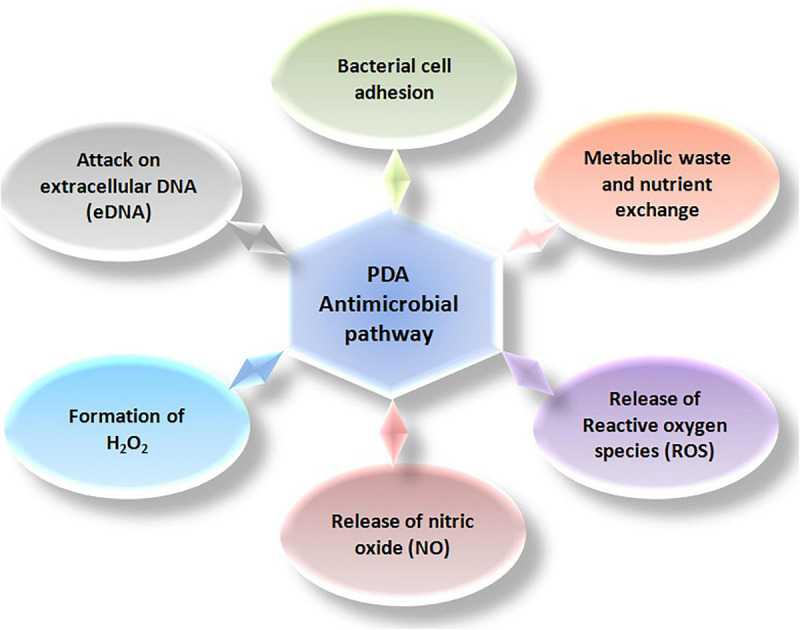
Representation of antimicrobial pathways essential for obstructing bacterial survivals.

Polydopamine has been illustrious for antibacterial and antifungal effects against several microorganisms (Figure 3). Su et al. (2016) have raised the scope of PDA by developing a simple shaking-assisted method to produce roughened polydopamine (rPDA) coatings at a variety of substrates. In the absence of an external antibacterial agent, the projected rPDA coatings displayed significantly enhanced antibacterial activity against Gram-positive and Gram-negative bacteria. Patel et al. (2018) have revealed that PDA coatings using different buffers could help in manipulating antibacterial activities, since the selection of the buffer could control the percentage of a particular functional group present in that PDA coating. Tris and sodium hydroxide-mediated PDA coating exhibited higher antibacterial activity as compared to that obtained using sodium bicarbonate and phosphate-buffered saline (PBS), which might be due to the presence of abundant hydroxyl groups on the surface of the coating. The findings of the study outlined the effect of different chemistries on the morphology and physicochemical properties of the PDA coatings that ultimately affected the antibacterial or antifouling properties. Zhou et al. have also suggested an alternative route to expedite PDA coating on the surfaces. High temperature with rapid shaking for 30 min produced the coated surfaces similar to properties exhibited by the surfaces coated for 24 h (Zhou et al., 2014).

**FIGURE 4:**
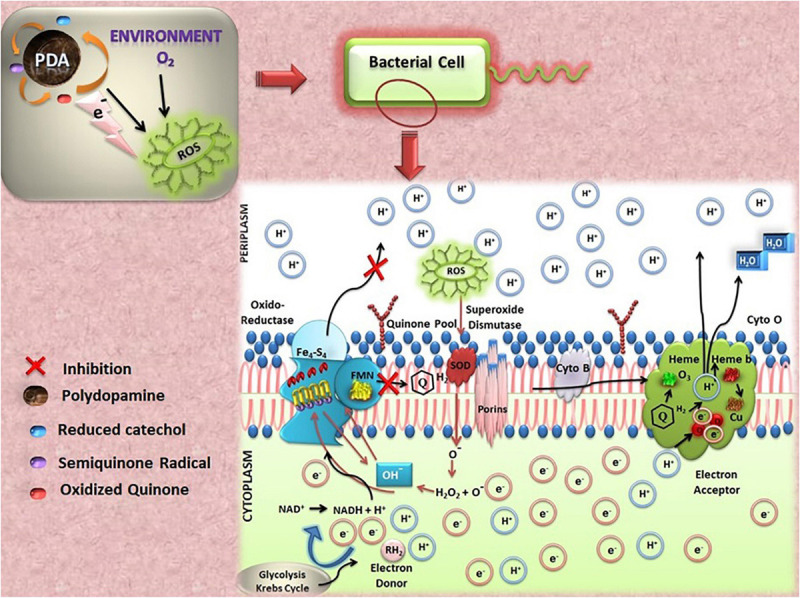
Representation of reactions occurred during electron transfer from polydopamine (PDA).

Polydopamine has been observed to possess redox-dependent properties, which make this material to perform as an antioxidant as well as a pro-oxidant. It can accept or donate electrons repeatedly to exhibit beneficial radical-scavenging properties as well as to generate ROS, which account for its antimicrobial properties (Liu and Huang, 2016; Lakshminarayanan et al., 2018; Liu et al., 2019). Its chemistry involves two electron transfer between quinone and hydroquinone structures. Catecholic moieties of PDA are responsible for donating electrons to oxygen molecule to generate hydrogen peroxide, which subsequently produces hydroxyl radical advocating the localized and instant antibacterial activity. These properties are greatly influenced by the presence of metal ions and near-infrared (NIR) irradiation. The series of reactions that occur during electron transfer (respiratory chain) is important for bacterial growths as well as PDA redox state, as respiratory chain inhibitors have been illustrated in Figure 4. Mechanistically, *in situ* formation of different forms of polydopamine acts as pro-oxidant/antioxidant owing to acceptance of electrons (e^–^) from the environmental oxygen and vice versa. The oxidized form of PDA attained by metal binding (Ag^+^, Au^3+^, Pt^4+^, Cu^2+^) inflates the generation of quinone moieties and affinity for binding other biomolecules to enhance microbial inhibitory effect (Liebscher, 2019). Electron-donating behavior of PDA produces detrimental pro-oxidant effects on bacterial survival via generation of reactive oxygen species (ROS) via conversion of environmental oxygen (O_2_) to superoxide radical (O_2_^–^). Generated ROS expedite the activity of superoxide dismutase (SOD; metalloenzyme), thus forming H_2_O_2_, which further produces OH. Combined effects of hydroxyl ion and the presence of NADH on Fe–S cluster along with cofactor FMN enrich bacterial cellular membrane with quinones (Quinone pool) (Larosa and Remacle, 2018). Synthesized quinone facilitates the formation of heme b and heme o_3_ for survival of prokaryotes. The transfer of electrons is an essential pathway for cellular respiration within microbes, especially bacteria. Herein, the pro-oxidant effect of PDA escalates ROS production and disrupts the chain of Fe–S cluster, thus leading to inhibition of formation of quinone and thus famine quinone pool. Irradiation and metal binding also alter the redox state behavior of PDA for exhibiting antimicrobial properties. PDA nanocoating exhibits a broad absorption range from visible to NIR, which can be tailored for various photothermal behaviors (Zou et al., 2020). The study by Lei et al. (2016) has reported that this PDA nanocoating possesses very high photothermal conversion properties upon NIR irradiation, which leads to the killing of all local microbes independently on genus. Medical-device-associated infections have been drastically increasing in the current scenario. Surface functionalization via altering surface topography (surface roughness/wettability/surface energy), chemical modification (smart surfaces), and multifunctional surfaces (nanoform layer/antimicrobial coating using antibiotics, antimicrobial peptides: AMPs) help in combating microbial adhesion using different pathways, i.e., contact killing mechanism, electrostatic repulsion between surface and bacterial cell, rupturing cellular membrane by nanopillars, hydrophilicity block action of bacterial cell adhesins (proteins, flagella, pilli), cationic/zwitterionic moieties repel negative charge strains and bind to + ve charge strain, thus rupturing cell membrane leading to leakage of cellular matrix (Ghilini et al., 2019).

## Endowing Antimicrobial Potential by PDA Fabricated Hydrophilic and Charged Surfaces

Alteration in the topographic pattern of various substrates (glass, polycarbonate, implants, pristine, etc.) helps in mitigating microbial adhesion, i.e., biofilm formation. This approach is well suited for coating medical implants and biomedical devices to combat microbial-associated infections. Nowadays, compromised surface geometry allows wide range of microbes for the attachment. Adhered microbes attract other microbial community for biofilm formation, and fouling tendency also supports biofilm formation. A recent study by Khanzada et al. revealed that cross-linked PDA-GO functionalized RO membranes exhibited bactericidal and antifouling properties. They demonstrated that hydrophilic behavior and smooth edges of the surface hindered bacterial adhesion and long-term survival. The rationale behind this outcome was the negative charge on the surface due to the presence of COOH, leading to electrostatic repulsion between the surface and Gram-negative strain and PDA-GO imparted synergistic effects on bacterial cellular damage via ROS production (Khanzada et al., 2020). In another study, an increase in surface roughness and hydrophilicity during surface modification with PDA-functionalized adhesions have been shown as important factors that expedite the binding of bacteria onto surfaces (Su et al., 2016); however, positively charged functional moieties in PDA cause lysis. Likewise, cationic polymer (chitooligosaccharide)-conjugated PDA-polyurethane (PDA-PU) membranes have shown significantly improved antibacterial activity as compared to PDA-PU membranes, which showed marginal enhancement in the properties over native PU membranes (Luo et al., 2017). In yet another report, Ahmed et al. have demonstrated the importance of hydrophilic behavior of PDA-functionalized polysulfone (PSF) substrate enriched with hydrophilic natural organic matter (NOM) for combating microbial adhesion. Herein, microbial adherence has been blocked by inhibiting interaction between cell surface adhesins, i.e., pilli and flagella and PDA-functionalized surface (Ahmed et al., 2018). A summary of the important PDA-based systems along with their mechanism of action on the microbes is depicted in Table 1.

**TABLE 1:** Summary of some of the polydopamine (PDA)-associated nanostructures and their bacterial inhibitory mechanism.

**S.No.**	**PDA conjugated ligands**	**Nanoform**	**Bacterial inhibition pathway**	**References**
1	PEG or sulfobetain@PDA(Ag)@substrate Substrate:TiO_2_, glass, Au, Nitinol alloy, PDMS, PS	Nanocoating	⊗ bacterial adsorption on substrate due to dense tightly bound water layer	Liu and Huang, 2016
2	PDA@substrate Substrate: glass, stainless steel, plastic, gauze	Nanocoating	⊘ bacterial cell surface	Su et al., 2016
3	NO@PDA hollow nanoparticles	Nanoparticles (Spherical)	NO release, **↑** nitrosative and oxidative stress within bacteria	Park et al., 2016
4	NO@PDA@substrate and NO@PEG@PDA@substrate	Nanocoating	Release NO and ⊗ bacterial adherence on surface	Sadrearhami et al., 2019
5	Ag@PDA@Ti	Nanocoating	ROS production, ⊘ membrane integrity	Zhou et al., 2020
6	Cu@Ag@PDA@PET fabric	Nanocoating	Interaction of Cu NPs with bacterial cell wall followed by rupturing	Wang K. et al., 2020
7	PDA@Ag-PVP NPs	Nanocoating	ROS mediated bacterial membrane damage	Niyonshuti et al., 2020
8	Ag@PDA@polyamide	Nanocoating	⊘ metabolic exchange through cell membrane thus cause cell death	Yang et al., 2016
9	Ag@PDA@PSU membrane	Nanocomposite layer	⊘ bacterial cell integrity	Tang et al., 2015
10	PLA-Au@PDA@Ag nanofibers	Nanofibers	⊘ DNA replication and bacterial intracellular processes and cell lysis	Zhang et al., 2019
11	Cu/Ag-PDA nanohybrids	Nanoparticles	**↑** in ROS level, membrane damage, ⊗ nutrient intake and membrane enzymatic/protein activities, cell death	Yeroslavsky et al., 2016
12	Colistin(Ag)@PDA	Hybrid Nanospheres	Synergistic effects of Colistin and Ag^+^. Membrane-damaging bactericidal activity. Adherence to membrane followed by generation of pores	Ran et al., 2020
13	Ag@PDA@PEEK implant material	Nanocoating	Synergistic effect for Inhibiting bacterial membrane sulphur containing enzymes, thus ⊗cellular respiration and cell death	Gao et al., 2017
14	Ag@PDA@sericin-PVA film	Nanocoating	Interaction with bacterial membrane followed by penetration by Ag ions	Cai et al., 2017
15	Ag@PDA@TPU porous membrane	Nanocoating	**↑**DNA damage, ⊗respiratory enzyme followed by cell death	Liu M. et al., 2018
16	Col@Ag@PDA@PP 3D scaffold	Nanofibrous scaffold	**↓**nutrient reservoir for bacteria and ⊗ migration thus perform killing effect	Qian et al., 2019
17	Hydrophobic silica@PDA@fabric	Nanocomposite layer	Charge dependent cytoplasmic membrane disruption, surface roughness and hydrophobicity interfere bacterial cellular communication and causes cell death	Song et al., 2019
18	γ-MG@PDA@contact lenses and AMP@PDA@contact lenses	Nanofilm	Bacterial membranolytic action, **↑** surface roughness ⊗ bacterial adhesion	Dhand et al., 2020
19	Cefotaxime@PDA@Ti implants	Nanocoating	Prevent adhesion and proliferation of bacteria	He et al., 2014
20	CS@Ag@HA@PDA@Ti implants CS-chitosan; HA-hydroxyapatite	Hybrid nanocoating	⊗ DNA replication, electron transport chain (ETC), and **↑** membrane permeability and leak intercellular constituents.	Li et al., 2016
21	Ag@PDA@Ti implants	Nanocoating	Ag ions interfere with cell division, generating reactive Oxygen species, induce stress in microorganisms, DNA damage, and finally cell death	Choi et al., 2019
22	Ag@PDA-CS@Ti	Nanocoating	Anti-adhesion activity of PDA-CS surface and antibacterial activity of Ag NPs	Wang B.B. et al., 2020
23	Cu^2+^@PDA@Ti	Nanocoating	Copper ions generate ROS by redox activities, interruption in the protein assembling and metabolism process followed by cell death	[Bibr B41][Bibr B124]
24	Ag@PDA@TiO_2_@Ti	Nanocoating	Ag ion mediated increase of intracellular stress and thus apoptosis	Jia et al., 2016
25	Ag@PDA@TiO_2_	Nanocoating	Ag mediated bacterial cell membrane cracking, protein denaturation, DNA damage and killing	Gao et al., 2019
26	Ag@PDA@TiO_2_@Ti substrate	Nanorods	⊗ bacterial cell wall synthesis and protein denaturation	Guan et al., 2019
27	RGD@PDA@MoS_2_@TiO_2_@Ti substrate	Nanocoating	**↑**cellular GSH oxidation, contact killing mechanism and laser induced leakage of internal matrix due to hyperthermia in bacteria causes cidal effects	Yuan et al., 2019
28	Sulfobetain@PDA(Cu)@ Zirconia disk	Nanocoating	Radical-mediated impairing intracellular bacteria	Fan et al., 2018
29	Ag@PDA@g-C_3_N_4_ scaffold	Nanosheets	Photogenerated electrons form ⋅OH, O_2_^–^ destruct biomolecules thus **↑** bacterial cell death	Wu Y. et al., 2018
30	Ag@PDA@Graphene oxide matrix	Nanocomposite	Interfere Cell permeability, ATP production, leak RNA thus kills bacteria	Liao et al., 2019
31	Ag@PDA@MoS_2_ sheet	Nanosheets	Photothermal induced cell membrane disruption and bactericidal effect.	Yuwen et al., 2018

***↑**: Increasing; **↓**: Decreasing;⊗ : Inhibition; ⊘ : Disruption/Interfere; **⋅OH:** Hydroxyl radical; **O_2_⋅**: Superoxide radical.*

## Cationic Charge Enriched PDA-Fabricated Surfaces: Effect on Antimicrobial Properties

Chemical moieties used for surface modifications such as cationic polymers, antimicrobial peptides (AMPs), zwitterionic polymers, and quaternary ammonium compounds (QAC) have been employed to introduce antifouling, antimicrobial, and antibiofilm properties, and the resulting surfaces have been used for combating device-associated infections (Ghilini et al., 2019). Gram-negative bacterial strains specifically adhere to cationic surfaces for longer time period, leading to penetration of bioactive molecules into the cell membrane via different transporter and porins. These frontrunners advocate for hindering series of metabolic activities essential for bacterial survival. On the other hand, Gram-positive bacterial community repels surfaces due to electrostatic repulsion, thus unable to promote biofilm formation. PDA has been proven as a versatile biopolymer for surface functionalization in conjugation with various positively charge compounds, as shown by Yao et al. (2018) in the fabrication of zwitterionic polysulfone surface to mitigate biofouling process and microbial adhesion. The approach helped in designing and developing smart surfaces that could inhibit biofilm formation, thus decreasing biofilm-associated infections. A recent study by Zhou et al. scrutinized the activity of cocktail functional polymers, cationic monomer, and cross-linker deposited on the catheter surface. These combinations inflated surface hydrophilicity as well as cationic charge, expediting biofilm inhibition (Zhou et al., 2018).

A review by Jia et al. (2019) has mentioned about the role of PDA-based coatings to improve the antimicrobial and osseointegration of orthopedic implants. The adhesive character of PDA has been used to attach the antimicrobial enzyme lysostaphin, covalently, to various surfaces to produce antibacterial and antibiofilm interfaces (Yeroslavsky et al., 2015). An easy, two-step, shaking-assisted PDA coating technique has been suggested to create antimicrobial polypropylene (PP) mesh having the capability to produce H_2_O_2_ (Forooshani et al., 2019). The coating was found to be more effective against Gram-negative bacteria, while Gram-positive bacteria showed a resistance. However, the generation of a higher amount of H_2_O_2_ as well as longer exposure time was found to be enough to destroy Gram-positive bacteria. Aminoglycoside conjugates of PDA have been evaluated for antimicrobial potency against different bacterial strains (clinical as well as resistant ones) e.g., PDA–kanamycin, PDA–gentamicin, and PDA–neomycin nanoconjugates (Singh et al., 2020). PDA deposited onto the polydimethylsiloxane (PDMS) surface has been used to tether synthetic antimicrobial cysteinylated tryptophane–arginine-rich peptide (CWFWKWWRRRRR-NH_2_) (CWR11) that displayed antimicrobial functionality over 21 days on catheter-relevant surfaces to combat catheter-associated urinary tract infections (Lim et al., 2015). Similarly, immobilization of liposomal amphotericin B (LAmB) on a PDA-coated PDMS surface, a material commonly used in the manufacturing of urinary catheters, was shown to impart the ability to resist *Candida albicans* colonization (Alves et al., 2019).

Antifouling surfaces composed of a zwitterionic copolymer, made up of 2-methacryloyloxyethyl phosphorylcholine and dopamine methacrylamide followed by covalent codeposition on polyethylenimine (PEI)/PDA surfaces, showed antibacterial activity against Gram-negative and Gram-positive bacteria along with adsorption resistance to bovine serum albumin after *in situ* deposition of AgNPs (Asha et al., 2018).

A material surface, composed of PDA-coated Fe_3_O_4_ nanoparticles followed by covalent tethering of barbituric acid, has been developed. Postreaction with sodium hypochlorite generated N-halamine groups on the imide functions of the barbituric acid, and the resulting surface displayed excellent antimicrobial activity against both Gram-positive and Gram-negative bacteria. These bifunctional nanoparticles with excellent antibacterial as well as magnetic properties not only destroyed targeted bacterial colonies but could also be recovered by applying an external magnetic field (Akter et al., 2018). In a similar type of study, N-halamine functions have been generated on PDA surface, and the resulting surface has showed significantly higher antibacterial activity toward Gram-positive and Gram-negative bacteria within a brief contact time as compared to the activity displayed by the only PDA-coated substrate (Chien et al., 2020).

The contribution of PDA to provide stability as an adhesive and antibacterial activity has been validated during evaluation of PDA/alginate/Fe_3_O_4_ hydrogel beads. The coating of PDA on to Alg/Fe_3_O_4_ beads not only significantly increased the stability in different pH, hydrophilicity, and elasticity but also performed strongly against bacterial strains (Matai et al., 2019). These beads have been demonstrated to be used repeatedly as antibacterial agents.

Polydopamine coatings have been employed on the surface of satellite telemetry tags, with coating conditions tailored to generate varying amounts of hydrogen peroxide. The coating displayed the ability to diminish the adhesion of *E. coli* and *Psychrobacter cryohalolentis* and, therefore, has the potential to decrease the chances for tissue infection at the tag implant site (Tyo et al., 2019).

Nitric oxide at low concentration affects cellular signaling within biofilms. Park et al. (2016) and Adnan et al. (2018) have reported PDA nanoparticles with diazeniumdiolate groups that could deliver nitric oxide (NO) for antibacterial therapy with negligible toxicity. A technology, using catecholamine to coat surfaces of body-implantable materials having diazeniumdiolate groups that could supply NO *in vivo* and that could be used without triggering cytotoxicity, has been patented (Kim et al., 2017). Further, PDA films with low-fouling and NO-releasing capabilities containing both diazeniumdiolate and polyethylene glycol (PEG) functionalities with NO release capability to over 48 h have been reported and showed the ability to inhibit the attachment of a multidrug-resistant strain and efficiently destroy the biofilm (Sadrearhami et al., 2019). Since catechol groups in PDA provide the ability to coordinate metal ions, Cu^2+^-loaded PDA coatings were able to catalyze NO release (Zhou et al., 2020).

The mechanism suggested in the synthesis of PDA/copper-doped calcium silicate (Cu-CaSil) bioactive hydrogel validated the coordination between PDA and Cu^2+^ ions. The projected complex showed enhanced photothermal performance of the hydrogel as observed by mass extinction coefficient profile and improved bioactivity. Laser irradiation of this hydrogel showed an excellent antibacterial activity. Stronger NIR absorption has been attributed to the presence of PDA polymer and Cu-CaSil powder, two hydrogel components, both possessing photothermal property, and the right PDA/Cu ratio leads to a number of tetracoordinated structures in PDA–Cu complex responsible for enhancing absorption intensity. Since heat treatments are known to kill bacteria, the photothermal effect of the composite hydrogel PDA/Cu-CaSil with synergistic antibacterial function of Cu^2+^ ions created unique “hot ions effect” by heating Cu^2+^ ions through laser irradiation (Xu et al., 2020). Further, Cu/Ag/PDA/PET fabrics have been shown to possess good antibacterial property against *E. coli* (∼99%) due to the adhesive ability of PDA and its strong binding to Cu NPs. The self-polymerization of dopamine under alkaline conditions forming PDA nanoparticles assisted a change in morphology of PET fabrics. The catechol moieties and amine functional groups in PDA reduced Ag^+^ to Ag NPs that were fastened on fiber surface via chelation between PDA and Ag^+^ ions. These Ag NPs, as catalytic seeds, facilitated deposition of Cu NPs on the surface of fabrics through chemical copper plating. Thus, PDA, functioning as template, expedited Cu NPs deposition onto the surface of fabric that displayed killing of most of the adhered bacteria (Wang K. et al., 2020).

The antimicrobial activity of Ag implants is mainly attributed to binding of Ag^+^ to thiol groups present in bacterial enzymes. Its use has been exploited with many PDA-assisted coatings (Yun’an Qing et al., 2018). Ag-coated PDA microspheres have been used to kill *Staphylococcus aureus* cells due to the elevated ROS level (Guo et al., 2019). The abundance of catechol and amine groups on the surface of PDA particles are the active sites for *in situ* reduction in the silver precursors, and the formed AgNPs can be fixed there; Wu C. et al. (2015) used [Ag(NH_3_)_2_]^+^ ions as a precursor for polydopamine-assisted electroless Ag metallization, which showed an excellent antibacterial performance against *Escherichia coli* and *S. aureus*.

The PDA coatings incorporated with Ag ions illustrate an attractive approach, as the combination facilitates interactions with biological system without cytotoxicity and exhibits antibacterial capability as well. The synergistic effects between PDA coating and AgNPs have been highlighted in a study based on X-ray photoelectron spectroscopy (XPS) and Fourier-transform infrared spectroscopy (FTIR) analysis by Niyonshuti et al. (2020). The study indicated that the PDA coating becomes thicker as the PDA deposition time is increased. The coordination between Ag and catechol groups on the PDA coating was found to significantly increase the potency of AgNPs against *E. coli*. Thus, a PDA coating is observed to play a significant role in enhancing the antimicrobial properties of AgNPs. The result indicated that catechol-rich PDA coating resulted in an increased ROS generation and significant damage to the bacterial membrane. The reducing catechol groups of PDA have further been explored to form well-dispersed AgNPs on different substrates for controlling biofouling and as antimicrobial reverse osmosis membranes, which, in turn, expose the potential of PDA to modify surface chemistries and morphological features of the membranes (Tang et al., 2015; Yang et al., 2016). Since the catechol groups in the PDA films chelate with Ag, the *in situ* reduction in Ag^+^ to Ag^*o*^ and binding of Ag^*o*^ to N- and O-sites in PDA film produce seed precursor, which helps in building up of AgNPs on the central venous catheters (CVCs) surface, demonstrating significant antimicrobial potency and appropriate biological safety (Wu K. et al., 2015).

A bio-nanocomposite coating using the mycogenerated AgNPs and PDA has been reported to display antibiofilm activity on biofilms of multidrug-resistant *A. baumannii* used in central venous catheter (Neethu et al., 2020). AgNPs formed by reduction through catechol have been reported not to be sensitive to oxygen and so have a longer-lasting antimicrobial effect (Wu H. et al., 2018). Zhang et al. (2019) combined the antimicrobial effect of three materials to boost the potential of bio-coated PLA-Au@PDA@Ag nanofibers. The large surface area of the AuNPs in the fibers facilitated the enhancement of the cell penetration with simultaneous production of oxidative stress by the biological system, thereby exhibiting high antibacterial activity. PDA, with its antibacterial effects, too, acted as a binder for different interfaces with effective anchoring groups.

Metal-containing PDA-NPs are suggested to be highly microbicidal and display effective antibiofilm activity (Yeroslavsky et al., 2016). Besides this, sonochemically synthesized Ag–PDA NPs and Cu/Ag–PDA hybrid NPs with copper shell and Ag core have been demonstrated to convert the surface to antibacterial. The projected system also gets benefited from the partial contribution to the antimicrobial activity from the stable PDA-semiquinone and ROS generated by the metal–PDA NPs under physiological conditions as suggested by Yeroslavsky et al. (2016). The synergistic actions of two or more antibacterial drugs as nanohybrids have been established to be effective in the treatment of refractory bacterial infections. The colistin-loaded PDA nanospheres decorated with silver nanodots are reported by Ran et al. (2020) to exhibit powerful antibacterial and antibiofilm effects.

The metal-ion-reducing ability of catechol in PDA with consequently depositing the Ag nanoparticles has been utilized to support antibacterial coatings for poly(ether ether ketone) (PEEK) implants. The PEEK–PDA–Ag implant was reported to inhibit growth of *E. coli* and *S. aureus* on the surface and the surrounding bone tissue compared to PEEK without affecting osseointegration (Gao et al., 2017). Further, PDA and silk fibroin have been introduced to the porous PEEK surface to balance the biocompatibility and antibacterial ability of PEEK implant by Yan et al. (2018). The study reported a first time PDA-assisted *in situ* growth of AgNPs and immobilization of silk fibroin (SF)/gentamicin sulfate (GS) coating upon porous PEEK surface. The dual application of PDA, reducing Ag^+^ into AgNPs followed by firm fastening onto PEEK surface and biocompatibility serving osteogenesis, was presented as a synergistic bacteria killing ability in AgNP-incorporated SF/GS coating constructed upon porous PEEK surface (Yan et al., 2018). PDA synthesized in one step is reported by Cai et al. (2017) to act as both a metal ion chelating as well as reducing agent to synthesize, *in situ*, AgNPs on the sericin/poly(vinyl alcohol) (PVA) composite film, which displayed long-standing antibacterial activities. In another study, Liu M. et al. (2018) have demonstrated prominent antibacterial activity of a porous thermoplastic polyurethane (TPU) membrane coated with PDA NPs followed by a layering of nanosilver and its use as a profound antibacterial invasion dressing (i.e., wound dressing) with high biocompatibility for wounds caused by clinical and antibiotic-resistant bacteria. Using mussel-inspired PDA coating technology, a scaffold (PP–PDA–Ag–COL) was generated by Qian et al. in a multistep process. An electrospun poly (lactide-co-glycolide) (PLGA)/poly(epsilon-caprolactone) (PCL) matrix was first coated with PDA NPs followed by coating with AgNPs via *in situ* reduction to incorporate antibacterial and osteogenic properties. The resulting PP–PDA–Ag matrix was then coated with type I collagen (COL) to further improve the properties including biocompatibility of the scaffold. Collagen I coating not only enhanced the cytocompatibility but also resulted in the consistent release of silver ions over more extended periods. The tailored PP–PDA–Ag–COL structure maintained the three-dimensional interfiber architecture of the scaffold (Qian et al., 2019). The emergence of PDA thin layer on the AgNPs, which exhibit cytotoxicity, has been presented to be highly biocompatible with almost no toxic effects on the cells and that too without compromising on the optical characteristics (Yilmaz, 2020). Various groups have also developed diversified types of PDA-coated surfaces; for instance, PDA-dyed hair prevented the scalp from bacterial infection. Likewise, PDA-coated cotton fabric, on treatment with quaternized nanosilica followed by hexadecyltrimethoxysilane, produced a superhydrophobic and excellent antibacterial material with good washable properties (Song et al., 2019). The design and development of antimicrobial contact lenses is another area where PDA-based coatings have been tested against bacterial adhesion and formation of biofilm. Dhand et al. examined the covalent and non-covalent interactions of antimicrobial agents with PDA coating on contact lenses. The results showed that surface properties of the lenses remained unaffected, and the coating displayed excellent antimicrobial activity to prevent biofilm formation for longer duration without any side effects on ocular cells (Dhand et al., 2020). The current state of the art for polydopamine research has revealed PDA as a smart adhesive biopolymer that has the potential to deal with diversified medical as well as environmental challenges.

The incorporation of antimicrobial agents on otherwise corrosion-resistant Ti surfaces is one of the effective modifications to enhance the biological properties of the implants, especially useful for orthopedic implant applications. The study on Ti–PDA–cefotaxime sodium (Ti–PDA–CS) substrates by He et al. indicated that the CS retained its biological activity even after immobilization onto the surface. The conjugated surface exhibited excellent *in vitro* antibacterial activity (He et al., 2014). The combination of PDA with hydroxyapatite (HA), a naturally occurring mineral form, with the formula Ca_10_(PO_4_)_6_(OH)_2_, calcium-phosphate (CaP)-based material, imparts good mechanical strength as well as corrosion resistance on the surface of implant materials. The formation of CaP biominerals onto the PDA-coated surface grown by layer-by-layer model brings morphological change emphasizing strong affinity between CaP and PDA moieties. The chemistry in PDA-HA coatings works well for antibacterial resistance. A review on functionalization of PDA-HA with applications toward bone–tissue engineering has highlighted the antimicrobial resistance (Kaushik et al., 2020). *In vitro* studies of a hybrid coating composed of bioactive species, hydroxyapatite (HA)/chitosan (CS)/Ag matrix, an organic–inorganic hybrid, onto the surface of a PDA-modified Ti implant could control the release of silver ion for good self-antibacterial execution as well as good osteoinductive ability. The catechol groups of PDA are supported to accelerate the formation of HA crystals and bridge the binding strength between HA and Ti substrate (Li et al., 2016). PDA-Ag coating on Ti surface has also been testified by Choi et al. (2019) to inhibit the formation of biofilm, microbial colonization, and pathogenesis of gum disease in the mouth, i.e., retardation of microbial growth. Furthermore, CS/AgNPs coating has been reinforced by employing PDA as an intermediate bridging layer on the urinary catheter and Ti surfaces, a simple immersion method in acid solution that is reported to exhibit phenomenal refinement in preventing bacterial adhesion. The availability of hydroxyl groups in chitosan (CS) molecules to reinforce antimicrobial AgNPs and control the Ag ions release besides strong attachment by PDA worked for a durable and efficient antibacterial coating (Wang B.B. et al., 2020).

The PDA acts as bonding glue for coating metal ions on the substrates. Cu(II) ions immobilization through PDA chelation on Ti surface for implant materials is reported to display antibacterial property (He et al., 2015). Wang et al. evaluated the antibacterial activity and osseointegration performance of Cu-deposited Ti substrates with PDA coating in the presence of bacterial infection both *in vitro* and *in vivo*. The Cu^2+^ ions affixed through coordination interaction inside the PDA coating showed sustained release of the metal ions. It was proposed that the released Cu^2+^ ions could effectively inhibit the bacteria in contact with the Ti–PDA–Cu substrate surfaces (Wang L. et al., 2017). Besides metal ion–PDA chemistry, there is interest to validate the effect of concentration of various metal ions on the antibacterial effect of Ti implants induced as self-assembled layer of adhesive PDA. The bacterial viability has been found to be inversely correlated with the ion concentration gradient of divalent Cu^2+^, Zn^2+^, and Sr^2+^ metal ions on PDA-coated Ti implant surface. The coated Ti implants may leach these ions to produce positive antibacterial efficacy. The chemical binding of metal ions with PDA has been suggested to control the release rate of the ion coating. Even though a strong Zn–PDA coordination bond in Zn coating slowed the release rate compared to Sr coating at the same concentration, 2% Zn coating and 10% Sr coating could inhibit bacterial growth of bacteria without any toxic effect on cells. The controversial results regarding the antibacterial activities of the PDA coatings suggest that the mechanism of bactericidal properties is still obscure (Kao et al., 2019).

The covalent adhesion and chelating talent of PDA have been exploited with bioactive and biocompatible titania coatings, TiO_2_, on Ti substrate and AgNPs, respectively, by Jia et al. (2016) for orthopedic coatings. Gao et al. also presented *in vitro* and *in vivo* bactericidal and antibiofilm activities of TiO_2_–PDA–Ag coating. The comparison in Ag release kinetics between the TiO_2_–Ag (without PDA) coatings and TiO_2_–PDA–Ag coating on Ti implants by electrochemical anodization synthesis showed a burst release in the former and a controlled-release pattern in the latter. Larger reduction of bacterial growth with overproduction of ROS observed in TiO_2_–Ag coating was recommended for the burst release, but the balance of cytotoxicity and the antibacterial effects lied in TiO_2_–PDA–Ag coatings (Gao et al., 2019). On the other hand, Guan et al. (2019) reported more durable and efficient antibacterial property in Ag–TiO_2_@PDA nanorods (NRDs) by hydrothermal synthesis than that of AgTiO_2_ NRDs, based on the synergistic effect of selective physical punctual and controlled release of silver ions. The ability of PDA to effectively transfer photoinduced electrons and protons and improve the photocatalytic activity has been applied in a three-dimensional Ag/TiO_2_/PDA nanofilm. PDA, as transformation interface, could support the photocatalytic mechanism to generate some ROS such as hydroxyl radical, hydrogen peroxide, and superoxides, which could cause bacterial inactivation. The study supported the future aspect of intermediate PDA layer as a favorable antibacterial coating on a wide variety of substrates such as plastic glass and metal alloy (Wen et al., 2020). Yuan et al. (2019) designed a functional MoS_2_/PDA–Arg–Gly–Asp–OH coating on Ti implants to inhibit *in situ* bacterial infection and mend osseointegration.

The material surface analyses of PDA onto the zirconia surface showed significant increase in cell adhesion and proliferation as compared with pristine zirconia. The coating suggested as peri-implant soft-tissue integration influenced human gingival fibroblasts and decreased adherent bacteria (Liu M. et al., 2015).

Silver nanoparticles developed using PDA coatings on rod-like mesoporous silica (SBA-15) gave SBA-15/PDA/Ag nanocomposites, which exhibited prolonged inhibitory effect on the growth of *E. coli*, *S. aureus*, and *Aspergillus fumigatus* (Song et al., 2018). A bioinspired coating with dual functioning antimicrobial, due to PDA and Cu^2+^ ions with a coating of zwitterionic sulfobetaine, via aza-Michael addition reaction, has been tested on commercial silicone-based urinary catheters (Fan et al., 2018). The hydrophobic–hydrophilic character, due to presence of amino, hydroxyl, and phenyl functions, good degradability, and antimicrobial properties of PDA have been exploited by Liu C. et al. (2018) to develop PDA-coated amorphous silica nanoparticle for hemorrhage control. PDA/SiNP displayed promising character for aggregating cells and inducing clotting. Compared to the commercial formulation, Celox, these coated nanoparticles shortened the blood clotting time to 150 s. These particles were found to achieve adequate hemostasis by accelerating coagulation and reducing blood loss during femoral artery and vein injury. PDA/SiNPs exhibited antimicrobial activity even for a longer duration with high hemocompatibility.

A PDA layer coating, synthesized by the H_2_O_2_/horseradish peroxidase method, has been found to reduce the cytotoxicity of carbon nanotubes (CNTs), enhance their dispersion, and endow better broad-spectrum photothermal antimicrobial activities in a gelatin-grafted dopamine/chitosan composite hydrogel (Liang et al., 2019). A PDA-based colloidal material synthesized through one-step dopamine polymerization by nitrogen-doped carbon dots has been reported to show an antibacterial effect against *S. aureus* through microorganism entrapment/ROS generation (Maruthapandi et al., 2019). The antibacterial activity of bio-photocatalyst on PDA-graphitic carbon nitride (g-C_3_N_4_) with uniformly dispersed AgNPs has been described (Wu Y. et al., 2018). Liao et al. used graphene oxide to form antibacterial Ag–PDA–RGO nanocomposites and evaluated their antimicrobial performance against Gram-positive and Gram-negative bacteria. The projected nanocomposites not only exhibited excellent antimicrobial activity but also opened newer avenues for a wide range of modern biomedical applications (Liao et al., 2019). Similarly, MoS_2_–PDA–Ag nanosheets demonstrated good antibacterial activity and eradicated *S. aureus* biofilms and wound infections (Yuwen et al., 2018).

## Conclusion and Future Prospective

This article makes a feature of the antibacterial role of PDA and the recent developments in the biomedical fields. The proficiency in robust adhesion of this polymer inspired by a structure similar to DOPA present in the amino acid sequence of mussel foot protein appeals to the researchers to try various substrates for surface modification. The self-polymerization of dopamine (DA) is still a mystery, but it has not stopped work on its functionalization, reacting quickly with amine and thiol-containing moieties.

The strong metal-chelating, covalent cross-linking, and redox capabilities have made it an innovative coating. Various forms of PDA nanomaterials, viz., NPs, microcapsules, and PDA hybrid nanospheres, hydrogels, and nanocomposites applicable for cell interfacing, biosensing, drug delivery, and tissue engineering, have been synthesized. As discussed for antimicrobial activities, PDA quandaries the cell membrane and barricades the cell surface, preventing diffusion of nutrients and wastes inside/out of the cell cytosol, leading to cell lysis. The positive charge on the functional groups in PDA is also responsible for lysis by contacting the bacterial cell wall.

The antimicrobial activity of PDA could be attributed to the presence of catechol, which forms semiquinone and quinones that get auto-oxidized in the presence of oxygen, generating ROS and subsequently preventing the growth of bacteria. However, the basis of different responses to different bacteria is still unclear. In some of the nanohybrid systems, the PDA role is restricted to stabilization of the adhesion of antimicrobial films or control the release of antimicrobial metal ions. PDA itself is being known for antibacterial activity due to regulated production of H_2_O_2_ during auto-oxidation; hence, optimization in its release is desired.

The versatile chemistry of biocompatible PDA warrants its study in medically relevant materials with or without passive and active agents, which prevent microbial biofilm formation and thus hold great potential for growth for diverse applications.

## Author Contributions

IS and SG compiled the manuscript. GD helped in editing and compilation. PK conceived the idea, edited, and supervised the task to completion. All authors contributed to the article and approved the submitted version.

## Conflict of Interest

The authors declare that the research was conducted in the absence of any commercial or financial relationships that could be construed as a potential conflict of interest.
